# Prevalence and extent of infarct and microvascular obstruction following a range of reperfusion techniques in ST-elevation myocardial infarction

**DOI:** 10.1186/1532-429X-15-S1-E63

**Published:** 2013-01-30

**Authors:** Jamal N Khan, Naveed Razvi, Anvesha Singh, Sheraz Nazir, Iain Squire, Gerry P McCann

**Affiliations:** 1 Cardiovascular Sciences, University of Leicester, Leicester, UK

## Background

Cardiac MRI (CMR) provides unique characterization of myocardial injury post acute ST-Elevation Myocardial Infarction (STEMI). It is the gold standard for non-invasive measurement of Infarct Size (IS) and tissue perfusion during STEMI. Microvascular obstruction describes suboptimal tissue perfusion despite restoration of flow in the infarct-related artery (IRA). IS and MVO are independent predictors of adverse remodelling and prognosis post STEMI. MVO is generally assumed to be related primarily to reperfusion.

There is a paucity of data on the prevalence and extent of MVO in clinical practice using a range of different reperfusion methods, and in particular in those without reperfusion. We hypothesize that the extent and presence of MVO are primarily related to the extent of ischaemia than reperfusion *per se*.

## Methods

88 acute STEMI subjects were recruited over a 26-month period. 14 subjects were routine clinical CMR examinations undertaken in non-reperfused, late-presenting patients (>12 hours onset of symptoms) to assess viability. Subjects underwent assessment on a Siemens Avanto 1.5T system. Left ventricular function and volumes were assessed using SSFP. 10 minutes after intravenous injection of gadolinium contrast (0.2 mmol/kg), delayed contrast-enhanced images were acquired using a segmented inversion-recovery gradient-echo pulse sequence. IS was defined as areas of hyperenhancement with signal intensity (SI) >50% of peak SI in the infarct core (Full-Width Half-Maximum technique). MVO was defined as hypointensity within areas of infarct.

## Results

There were no significant differences in age, proportion of STEMI with left anterior descending artery (LAD) IRA, or gender in each of the reperfusion groups (non-reperfused; all reperfused patients [combined lysis and PCI groups]; primary percutaneous angioplasty [PPCI]; thrombolysis; rescue PCI; late PCI [>12 hours]). Time from onset of symptoms to reperfusion was significantly longer in late PCI patients (p<0.0001). There was a trend towards larger IS in late PCI and rescue PCI, followed by those with no reperfusion. There was a trend towards larger extent of MVO in late PCI followed by no reperfusion and rescue PCI. Prompt thrombolysis resulted in a trend towards smallest IS and significantly lower extent of MVO. Chi-squared analysis demonstrated a trend towards higher prevalence of MVO in non-reperfused STEMI (75.0% v 61.1%).

## Conclusions

There is a higher prevalence and extent of myocardial and microvascular injury (IS, MVO) in non-reperfused STEMI than in those undergoing timely reperfusion. MVO is not exclusive to reperfusion and may primarily represent the degree of ischaemic injury. This study suggests that rescue PCI and late PCI offer no reduction in IS or MVO compared with those undergoing no revascularisation.

## Funding

This study was funded by a British Heart Foundation research grant, with support from the University of Leicester Biomedical Research Unit.

**Table 1:** CMR results for the reperfusion strategies

CMR marker	Non Reperfused (n=16)	All reperfused combined (PCI+Lysis Groups, n=72)	PPCI (n=47)	Lysis (n=12)	Rescue PCI (n=8)	Late PCI (n=5)	Non v All Reperfused (p)	Non v PCI (p)	Non v Lysis (p)	Non v Rescue PCI (p)	Non v Late PCI (p)
IS (%LV mass)	23.72 (13.38-32.40)	21.28 (14.31-34.91)	20.67 (13.33-31.06)	18.73 (11.74-28.11)	34.25 (21.26-41.52)	39.16 (21.11-45.51)	0.965	0.693	0.307	0.142	0.117

MVO (%LV mass)	1.94 (0.15-5.08)	0.56 (0.00-3.22)	0.49 (0.00-3.28)	0.22 (0.00-0.99)	1.16 (0.00-4.61)	4.11 (0.95-11.12)	0.231	0.242	0.047	0.664	0.281

Age (years)	63.12 (17.32)	59.75 (11.98)	60.49 (12.30)	63.12 (17.33)	59.50 (12.47)	63.13 (17.33)	0.353	0.509	0.501	0.605	0.318

Sex (M:F ratio)	14:2	66:6	41:6	11:1	8:0	5:0	0.602	0.893	0.729	0.307	0.417

Time To Reperfusion (mins)	n/a	268.61 (275.67)	187.04 (111.94)	213.82 (152.04)	281.63 (91.24)	1135 (822-1365)	n/a	n/a	n/a	n/a	n/a

Culprit vessel (LAD:non-LAD ratio)	7:5 (n=12)	34:37 (n=71)	19:28 (n=47)	6:5 (n=11)	4:4 (n=8)	3:2 (n=5)	0.641	0.269	0.858	0.721	0.960

Normally distributed data expressed as mean (SD). Non-parametric data expressed as median (25th-75th quartile range) and analysed using Mann-Whitney analysis. Statistical significance taken at p<0.05.

